# Early Life Factors and Risk of Childhood Rhabdomyosarcoma

**DOI:** 10.3389/fpubh.2013.00017

**Published:** 2013-05-31

**Authors:** Anshu Shrestha, Beate Ritz, Simona Ognjanovic, Christina A. Lombardi, Michelle Wilhelm, Julia E. Heck

**Affiliations:** ^1^Department of Epidemiology, School of Public Health, University of California Los AngelesLos Angeles, CA, USA; ^2^Precision Health EconomicsLos Angeles, CA, USA; ^3^Division of Pediatric Epidemiology and Clinical Research, Department of Pediatrics, University of MinnesotaMinneapolis, MN, USA; ^4^Masonic Cancer Center, University of MinnesotaMinneapolis, MN, USA

**Keywords:** birth weight, prenatal care, pediatric, rhabdomyosarcoma

## Abstract

Although little is known about etiology of childhood rhabdomyosarcoma (RMS), early life factors are suspected in the etiology. We explored this hypothesis using linked data from the California Cancer Registry and the California birth rolls. Incident cases were 359 children <6-year-old (218 embryonal, 81 alveolar, 60 others) diagnosed in 1988–2008. Controls (205, 173), frequency matched on birth year (1986–2007), were randomly selected from the birth rolls. We examined association of birth characteristics such as birth weight, size for gestational age, and timing of prenatal care with all-type RMS, embryonal, and alveolar subtypes. Crude and adjusted odds ratios (ORs) and 95% confidence intervals (95% CIs) were estimated using logistic regression. In contrast to a previous study, we observed statistically non-significant association for embryonal subtype among high birth weight (4000–5250 g) children for term births [OR (95% CI): 1.28 (0.85, 1.92)] and all births adjusted for gestational age [OR (95% CI): 1.21 (0.81, 1.81)]. On the other hand, statistically significant 1.7-fold increased risk of alveolar subtype (95% CI: 1.02, 2.87) was observed among children with late or no prenatal care and a 1.3-fold increased risk of all RMS subtypes among children of fathers ≥35 years old at child birth (95% CI: 1.00, 1.75), independent of all covariates. Our finding of positive association on male sex for all RMS types is consistent with previous studies. While we did not find a convincingly positive association between high birth weight and RMS, our findings on prenatal care supports the hypothesis that prenatal environment modifies risk for childhood RMS.

## Introduction

Rhabdomyosarcoma (RMS) is the most common type of soft-tissue sarcoma among 0- to 19-year-olds, with the incidence rate highest among children of ages 0–4 (1). Embryonal and alveolar RMS are the two main histological subtypes, accounting for over 90 percent of cases in children under the age of five (2). Genetic aberrations associated with RMS include germline mutations in the *TP53* tumor suppressor gene, translocation of chromosomes 2 and 13 or 1 and 13, and abnormalities on chromosome 11p15 where the *IGF2* gene is located (3). Indeed, RMS is often characterized by overexpression of the gene encoding insulin-like growth factor 2 (*IGF2*). This can result from either loss of heterozygosity (LOH, observed in both subtypes) due to the loss of the maternal allele and duplication of paternal allele, or loss of imprinting (LOI) via hypomethylation of the maternal *IGF2* allele (observed in embryonal RMS; 4–7). The alveolar subtype is further characterized by translocations between chromosomes 2 or 1 and 13 (8). In addition, germline mutations of the *TP53* gene, also implicated in regulation of *IGF2* expression, are often associated with early onset of RMS (9–11).

Childhood cancers tend to have a relatively short lag time between exposure and disease onset (12). Therefore, early childhood disease such as RMS could be due to exposures during prenatal, early infancy periods, or even during gametogenesis. Although most RMS cases are sporadic in nature, suggesting *de novo* genetic and epigenetic alterations perhaps due to environmental factors, some cases (7–33%) have familial predisposition as observed in families with Li-Fraumeni syndrome (LFS) and neurofibromatosis (13, 14). Very little is known about environmental factors that may trigger such genetic and epigenetic alterations resulting in an increased susceptibility to these cancers in children. A few studies have linked RMS in children with early life factors such as parental preconceptional use of recreational drugs including marijuana, maternal history of stillbirths, prenatal X-ray exposure, higher maternal age at childbirth, parity, and high birth weight (14–17). In two studies that examined embryonal RMS separately from other subtypes, the latter four risk factors were of more importance for the embryonal subtype (14, 17).

Overexpression of the gene encoding *IGF2* due to LOI/LOH observed in RMS has been implicated in both fetal overgrowth disorders that predispose children to embryonal tumors (including RMS) and RMS tumor cell growth and motility (6, 18, 19). Thus, high birth weight, one of the manifestations of fetal overgrowth disorder, could be an early marker of the genetic or epigenetic abnormalities present in the *IGF2* pathway that increase childhood tumor susceptibility. Indeed, studies on Wilms tumor, another embryonal tumor, have shown an association with high birth weight (20). A recent study that examined childhood RMS cases using pooled data from five states, including 0- to 4-year-old cases for 1988–1997 in California, provided some evidence of higher RMS odds in larger birth weight and large size for gestational age infants, particularly for embryonal RMS (14). However, the study’s eligibility and matching criteria for case and control selection varied across the states. This study improves on the previous work by removing non-uniformity in sample selection, excluding controls who died prior to diagnosis time of frequency matched cases, and including 10 additional years of incident cases among 0- to 5-year-olds in California (diagnosed in 1988–2008). It first aims to examine whether high birth weight as measured by birth weight with and without adjustment for gestational age is predictive of RMS, particularly embryonal RMS. In addition, it aims to examine whether other factors reported on birth certificates may be related to RMS risk in a large and ethnically diverse population.

## Materials and Methods

### Study population

We conducted a population-based case-control study in California, which included all cases diagnosed between 1988, since the inception of the California Cancer Registry (CCR), and 2008 among children of ages 0–5 years. Using first name, last name, and date of birth, we were able to match 89% of these cancer cases to a California birth certificate (1986–2007). Controls, frequency matched on birth year, were randomly selected in 1:20 ratio for all California registered <6-year-old cancer cases from the California birth roll. We ensured that the controls were cancer free up to age six or at the age attained by 2008 if younger than 6 years. Cancer-related information including RMS diagnosis, histology, and year of diagnosis were obtained from the CCR, while prenatal and perinatal data including birth weight, gestational age, parental age, and demographic information were collected from birth registry records. Institutional review board approvals were obtained from the University of California Los Angeles, the California Committee for the Protection of Human Subjects, and the CCR.

The RMS cases were those assigned site group of IXa in the International Classification of Childhood Cancer, third edition (ICCC-3) and the subtypes were based on the following histological codes from the International Classification of Disease for Oncology, third edition (ICD-O-3): 8900/3 – RMS (unspecified), 8901/3 – pleomorphic RMS, 8902/3 – mixed type RMS, 8910/3 – embryonal RMS, 8912/3 – spindle cell RMS, 8920/3 – alveolar RMS, and 8991/3 – embryonal sarcoma (21). Here, we evaluated all histological types (“all-type RMS”) and embryonal and alveolar subtypes separately.

We started with a total of 364 RMS cases and 209,700 controls. After excluding 1,522 controls who died before age 6 years, 57 children with Down syndrome, and births with implausible or extreme birth weight or gestational age values (27 with <500 g, 166 with >5250 g, 106 with gestational age <20 weeks, and 2654 with >45 weeks), the final study population included 359 RMS cases and 205,173 controls.

### Data collection and variable definitions

The birth registry data included birth weight (grams), gestational age (days), sex of infant, date of birth, parental age at child birth, plurality (singleton, multiple birth), parity, maternal pregnancy history (any prior pregnancy termination, any prior stillbirths), prenatal care (present from the first trimester onward, started after the first trimester/no care), and maternal race/ethnicity (non-Hispanic white, Hispanic of any race, and others). Gestational age was calculated based on the interval from date of last menstrual period to birth. For children born in 2007, obstetric estimated gestational age was also available. Thus, for those without gestational age, the obstetric estimated gestational age was used where available. Size at gestation was assigned as small if birth weight was less than the 10th percentile and as large if greater than the 90th percentile of the birthweight standards for a given gestational age. The 10th and the 90th percentile values were obtained for each gestational week (20–45 weeks) by maternal race/ethnicity (non-Hispanic white, Hispanic of any race, black, Asian/Pacific Islander, and other) and child’s sex based on the total singleton live births in California between 1988 and 2006 using the method described by Alexander et al. (22). The large number of births in California during the study period (*n* = 10,134,074) allowed us to use Californian instead of US births as a standard; this standard is more likely to be relevant for our diverse study population. In order to study the role of socioeconomic status, we applied a census-based SES index which combines seven census-block group indicators from 2000 census data: education index, median household income, percent living 200% below poverty level, percent blue-collar workers, percent older than 16 years in workforce without job, median rent, and median housing value (23).

### Statistical analysis

We used logistic regression to examine, in crude and adjusted models, associations between both birth weight and measures of fetal growth at a given gestational age (term birth weight, birth weight by gestational age, and size at gestational age) and the three outcomes of interest (all-type RMS, embryonal RMS, and alveolar RMS). In addition, we examined associations with the following perinatal and demographic characteristics: parental age at child birth, child’s sex, plurality, parity, timing of prenatal care, maternal race/ethnicity, gestational age, and census-based SES. We also conducted trend tests for birth weight, gestational age, and parental age at child birth. The following covariates, selected based on literature, DAGs (directed acyclic graphs), and initial analysis, were included in all adjusted models: birth year, child’s sex, plurality, parity, maternal age, maternal race/ethnicity, and prenatal care. The SES variable was excluded from the final analysis and the results presented since the effect estimate of interest changed by less than 1% when adding this variable in the model. All analyses were carried out using SAS version 9.2 (SAS institute, Inc., NC, USA).

## Results

Among the 359 RMS cases, the majority – 218 (61%) – were embryonal RMS cases, 81 (23%) alveolar, and 60 (17%) other types or unspecified cases. Both case mothers and fathers were slightly older than control parents. Male children were over-represented among RMS cases overall (61%) as well as among embryonal and alveolar subtypes (Table 1). A little over half of the cases had US-born mothers (57%), while the rest were Mexican born (23%) and other foreign born (19%) mothers (data not shown). A similar distribution was observed for the control group as well. Maternal history of pregnancy terminations and stillbirths were similar across the control and case groups (data not shown). The remaining demographic and perinatal characteristics also were similar across the two groups (Table 1).

**Table 1:** **Frequency distribution of demographic and perinatal characteristics across cases and controls**.

Variable name	Embryonal (*n* = 218)	Alveolar (*n* = 81)	All-type RMS (*n* = 359)	Controls (*n* = 205173)
	
	Frequency (%)	Frequency (%)	Frequency (%)	Frequency (%)
**CHILD’S GENDER**				
Male	136 (62.4)	47 (58.0)	220 (61.3)	104677 (51.0)
Female	82 (37.6)	34 (42.0)	139 (38.7)	100496 (49.0)
**PLURALITY**				
Singleton	209 (95.9)	77 (95.1)	345 (96.1)	200043 (97.5)
Multiple birth	9 (4.1)	4 (4.9)	14 (3.9)	5130 (2.5)
**MATERNAL AGE AT CHILD BIRTH**				
<20	19 (8.7)	8 (9.9)	29 (8.1)	22298 (10.9)
20–29	104 (47.7)	42 (51.9)	184 (51.3)	107054 (52.2)
30–34	60 (27.5)	19 (23.5)	92 (25.6)	47617 (23.2)
≥35	35 (16.1)	12 (14.8)	54 (15.0)	28169 (13.7)
Missing	0 (0.0)	0 (0.0)	0 (0.0)	35 (0.0)
**MATERNAL RACE/ETHNICITY**				
Non-Hispanic white	92 (42.2)	25 (30.9)	141 (39.3)	74182 (36.2)
Hispanic of any race	81 (37.2)	36 (44.4)	143 (39.8)	92200 (44.9)
Other (black, Asian/PI, other)	44 (20.2)	19 (23.5)	73 (20.3)	37589 (18.3)
Missing	1 (0.5)	1 (1.2)	2 (0.6)	1202 (0.6)
**PATERNAL AGE AT CHILD BIRTH**				
<20	5 (2.3)	4 (4.9)	10 (2.8)	7937 (3.9)
20–29	86 (39.5)	29 (35.8)	137 (38.2)	85838 (41.8)
30–34	52 (23.9)	20 (24.7)	89 (24.8)	49426 (24.1)
≥35	61 (28.0)	23 (28.4)	99 (27.6)	48633 (23.7)
Missing	14 (6.4)	5 (6.2)	24 (6.7)	13339 (6.5)
**CENSUS-BASED SES^a^**				
1 (Lowest)	58 (26.6)	21 (25.9)	92 (25.6)	49005 (23.9)
2	42 (19.3)	18 (22.2)	70 (19.5)	47657 (23.2)
3	50 (22.9)	19 (23.5)	86 (24.0)	45984 (22.4)
4	26 (11.9)	10 (12.4)	48 (13.4)	33377 (16.3)
5 (Highest)	42 (19.3)	13 (16.1)	63 (17.6)	28513 (13.9)
Missing	0 (0.0)	0 (0.0)	0 (0.0)	637 (0.3)
**PREGNANCY HISTORY**				
Parity
Nulliparous	88 (40.4)	33 (40.7)	140 (39.0)	80776 (39.4)
One child	75 (34.4)	27 (33.3)	122 (34.0)	64082 (31.2)
Two children	33 (15.1)	11 (13.6)	57 (15.9)	34565 (16.9)
Three or more children	22 (10.1)	10 (12.4)	40 (11.1)	25750 (12.6)
**PRE- AND POST-CONCEPTIONAL INFORMATION**				
Prenatal care
First trimester onward	173 (79.4)	59 (72.8)	277 (77.2)	162462 (79.2)
Started after first trimester/no care	45 (20.6)	22 (27.2)	79 (22.0)	40054 (19.5)
Missing	0 (0.0)	0 (0.0)	3 (0.8)	2657 (1.3)
Gestation length (weeks)
20–32	6 (2.8)	2 (2.5)	9 (2.5)	3655 (1.8)
33–36	16 (7.3)	4 (4.9)	27 (7.5)	16444 (8.0)
37–42	175 (80.3)	69 (85.2)	292 (81.3)	168524 (82.1)
43–45	13 (6.0)	3 (3.7)	17 (4.7)	8123 (4.0)
Missing	8 (3.7)	3 (3.7)	14 (3.9)	8427 (4.1)
Birth weight (in grams)
500–1499	2 (0.9)	1 (1.2)	3 (0.8)	1617 (0.8)
1500–2499	9 (4.1)	4 (4.9)	20 (5.6)	10367 (5.1)
2500–3999	179 (82.1)	70 (86.4)	297 (82.7)	171111 (83.4)
4000–5250	28 (12.8)	6 (7.4)	39 (10.9)	22078 (10.8)
Size for gestational age^b^
Small for gestational age (SGA)	23 (10.6)	9 (11.1)	40 (11.1)	20618 (10.1)
Normal size	164 (75.2)	64 (79.0)	273 (76.0)	156142 (76.1)
Large for gestational age (LGA)	23 (10.6)	5 (6.2)	32 (8.9)	19986 (9.7)
Missing	8 (3.7)	3 (3.7)	14 (3.9)	8427 (4.1)

*^a^Created using method described by Yost et al. (23), which is based on seven census-block group indicators: education, median household income, % living <200% poverty level, % blue-collar workers, % >16-year-olds in workforce without job, median rent, and median housing value. ^b^Standard for race/ethnicity and sex generated from the total births in California, 1988–2006*.

Our adjusted point estimates suggested a small statistically non-significant increase in risk of embryonal RMS for high birth weight (4000–5250 g) infants who were born at term and in all infants when adjusting for gestational age [odds ratio (OR) (95% CI): 1.28 (0.85, 1.93) and 1.21 (0.81, 1.82) respectively], but not for alveolar RMS or all-type RMS cases (Table 2). Trends toward positive associations between embryonal RMS and high birth weight was also observed for all the other measures of growth we explored, i.e., large size for gestational age, birth weight without adjustment for gestational age, a continuous per 500-g increase in birth weight measure with and without adjustment for gestational age. However, all 95% CIs were wider and again included the null value commensurate with no association (Table 2). The Cochran–Armitage trend test for both birth weight and gestational age showed no indication of linear association (2-sided *p*-values: 0.83 and 0.94 respectively).

**Table 2:** **Unadjusted and adjusted logistic regression outcomes for birth weight and size at gestational age**.

Variable name	Unadjusted^a^			Adjusted for covariates^b^		
	Embryonal (*n* = 218)	Alveolar (*n* = 81)	All-type RMS (*n* = 359)	Embryonal (*n* = 218)	Alveolar (*n* = 81)	All-type RMS (*n* = 359)
	
	OR (95% CI)	OR (95% CI)	OR (95% CI)	OR (95% CI)	OR (95% CI)	OR (95% CI)
**ALL BIRTH WEIGHT (IN GRAMS)**						
<2500	0.88 (0.48, 1.62)	1.00 (0.41, 2.49)	1.10 (0.72, 1.69)	0.75 (0.39, 1.44)	0.80 (0.30, 2.16)	1.00 (0.64, 1.58)
2500–3999	Reference
4000–5250	1.21 (0.81, 1.80)	0.68 (0.30, 1.57)	1.02 (0.73, 1.43)	1.16 (0.78, 1.74)	0.60 (0.24, 1.50)	0.96 (0.68, 1.36)
**TERM BIRTH WEIGHT (IN GRAMS)**						
<2500	1.12 (0.46, 2.74)	1.08 (0.26, 4.40)	1.38 (0.71, 2.52)	0.92 (0.37, 2.33)	0.83 (0.19, 3.56)	1.13 (0.59, 2.19)
2500–3999	Reference
4000–5250	1.32 (0.88, 1.98)	0.58 (0.23, 1.45)	1.07 (0.76, 1.51)	1.28 (0.85, 1.93)	0.62 (0.25, 1.55)	1.05 (0.74, 1.48)
**ALL BIRTH WEIGHTS ADJUSTED FOR GESTATIONAL AGE (IN GRAMS)**						
<2500	0.81 (0.40, 1.64)	1.22 (0.42, 3.49)	1.14 (0.70, 1.87)	0.69 (0.33, 1.44)	0.99 (0.33, 3.02)	1.04 (0.62, 1.75)
2500–3999	Reference
4000–5250	1.26 (0.84, 1.88)	0.58 (0.23, 1.43)	1.03 (0.74, 1.45)	1.21 (0.81, 1.82)	0.60 (0.24, 1.50)	1.00 (0.71, 1.40)
**PER 500-GRAM INCREASE IN BIRTH WEIGHT FOR:**						
All births	1.03 (0.91, 1.16)	0.98 (0.81, 1.19)	1.01 (0.92, 1.11)	1.04 (0.91, 1.17)	0.99 (0.81, 1.22)	1.01 (0.91, 1.11)
Term birth (≥37 weeks)	1.06 (0.92, 1.23)	0.94 (0.74, 1.19)	1.04 (0.92, 1.16)	1.07 (0.92, 1.25)	0.98 (0.76, 1.26)	1.05 (0.93, 1.18)
Birth weight adjusted for gestational age	1.05 (0.92, 1.19)	0.91 (0.73, 1.14)	1.01 (0.91, 1.12)	1.05 (0.92, 1.20)	0.94 (0.75, 1.19)	1.00 (0.90, 1.12)
**SIZE FOR GESTATIONAL AGE^c^**						
Small for gestational age (SGA)	1.06 (0.69, 1.64)	1.07 (0.53, 2.14)	1.11 (0.80, 1.55)	1.01 (0.65, 1.58)	0.98 (0.48, 2.00)	1.07 (0.76, 1.50)
Normal size	Reference
Large for gestational age (LGA)	1.10 (0.71, 1.69)	0.62 (0.25, 1.54)	0.92 (0.64, 1.32)	1.14 (0.73, 1.76)	0.51 (0.18, 1.40)	0.91 (0.62, 1.32)

*^a^Adjusted for birthyear. ^b^Adjusted for the following variables in addition to gestational age where mentioned: birth year, child sex, plurality, maternal race/ethnicity, maternal age, parity, and prenatal care. ^c^Standard for race/ethnicity and sex generated from the total births in California, 1988–2006*.

In terms of other perinatal characteristics, we found statistically significant increased risk for all-type RMS and embryonal RMS among male compared to female children, and for alveolar RMS among those with late initiated or no prenatal care [OR (95% CI): 1.52 (1.23, 1.89), 1.60 (1.22, 2.11), and 1.71 (1.02, 2.87) respectively] after adjusting for all covariates. We found a very small increase in risk for all-type RMS with each year increase in both maternal and paternal age at childbirth [OR (95% CI): 1.02 (1.00, 1.04) and 1.02 (1.00, 1.04) respectively]. Examining categorical variables, we observed a 32% increase in risk of all-type RMS for children with 35 years or older fathers [OR (95% CI): 1.32 (1.00, 1.75)]. We also observed approximately 30% increase in risk in mothers 30 years or older and fathers 35 years or older for embryonal RMS cases, albeit statistically non-significant (Table 3). The Cochran–Armitage trend test for parental ages across the three outcomes confirmed a linear trend of paternal age for all-type RMS (2-sided *p*-value: 0.03), but not for embryonal and alveolar subtypes separately (*p*-values: 0.09 and 0.36 respectively). As for maternal age, none of the outcomes showed a linear trend (*p*-values: 0.08 – all-type RMS, 0.06 – embryonal RMS, 0.72 – alveolar RMS). In addition, our data suggest a possible 60% risk increase for multiple compared to single births for all-type RMS [OR (95% CI): 1.60 (0.94, 2.75)]. Furthermore, delivery at late gestational ages (43–45 weeks) may increase embryonal RMS risk by as much as 56% [OR (95% CI): 1.56 (0.89, 2.74)], but the 95% CIs included the null values for these estimates indicating statistically non-significant. In contrast, children of Hispanic mothers appeared to have about a 20% reduced risk for embryonal [OR (95% CI): 0.78 (0.57, 1.08)], but not for alveolar RMS [OR (95% CI): 1.10 (0.64, 1.89)]. However, the 95% CI for adjusted models included the null value (Table 3). We further compared the children of Hispanic mothers born in Mexico and the US to US-born white mothers separately by embryonal RMS status, and the estimates for the two groups were similar [OR (95% CI): 0.75 (0.51, 1.09), 0.68 (0.43, 1.10), respectively].

**Table 3:** **Unadjusted and adjusted logistic regression outcomes for perinatal characteristics**.

Variable name	Unadjusted^a^			Adjusted for covariates^b^		
	Embryonal (*n* = 218)	Alveolar (*n* = 81)	All-type RMS (*n* = 359)	Embryonal (*n* = 218)	Alveolar (*n* = 81)	All-type RMS (*n* = 359)
	
	OR (95% CI)	OR (95% CI)	OR (95% CI)	OR (95% CI)	OR (95% CI)	OR (95% CI)
**CHILD’S CHARACTERISTICS**						
Child’s gender
Male	1.59 (1.21, 2.09)	1.33 (0.86, 2.07)	1.52 (1.23, 1.88)	1.60 (1.22, 2.11)	1.28 (0.82, 1.99)	1.52 (1.23, 1.89)
Female	Reference
Plurality
Singleton	Reference
Multiple birth	1.70 (0.87, 3.31)	1.93 (0.71, 5.28)	1.58 (0.92, 2.69)	1.70 (0.87, 3.32)	2.11 (0.76, 5.81)	1.60 (0.94, 2.75)
Gestation length (in weeks)
20–32	1.58 (0.70, 3.57)	1.34 (0.33, 5.46)	1.42 (0.73, 2.76)	1.45 (0.63, 3.34)	1.05 (0.25, 4.46)	1.30 (0.66, 2.55)
33–36	0.94 (0.56, 1.57)	0.59 (0.21, 1.61)	0.95 (0.64, 1.41)	0.89 (0.52, 1.50)	0.51 (0.18, 1.42)	0.89 (0.60, 1.34)
37–42	Reference
43–45	1.53 (0.87, 2.68)	0.97 (0.31, 3.09)	1.22 (0.75, 1.99)	1.56 (0.89, 2.74)	0.97 (0.30, 3.08)	1.23 (0.75, 2.01)
**MATERNAL CHARACTERISTICS**						
Maternal age at child birth
<20	0.88 (0.54, 1.43)	0.92 (0.43, 1.96)	0.76 (0.51, 1.12)	0.85 (0.51, 1.41)	0.83 (0.38, 1.81)	0.73 (0.49, 1.10)
20-29	Reference
30-34	1.31 (0.95, 1.80)	0.99 (0.57, 1.69)	1.12 (0.87, 1.44)	1.33 (0.96, 1.84)	1.01 (0.57, 1.78)	1.13 (0.88, 1.47)
≥35	1.31 (0.89 (1.92)	1.00 (0.53, 1.91)	1.11 (0.82, 1.51)	1.30 (0.87, 1.95)	1.09 (0.56, 2.14)	1.10 (0.80, 1.51)
Per 1-year increase	1.02 (1.00, 1.05)	1.00 (0.97, 1.04)	1.02 (1.00, 1.04)	1.02 (1.00, 1.05)	1.01 (0.97, 1.05)	1.02 (1.00, 1.04)
Maternal race/ethnicity
Non-Hispanic white	Reference
Hispanic of any race	0.72 (0.53, 0.97)	1.06 (0.63, 1.77)	0.81 (0.64, 1.02)	0.78 (0.57, 1.08)	1.10 (0.64, 1.89)	0.85 (0.66, 1.08)
Other	0.95 (0.66, 1.37)	1.41 (0.78, 2.57)	1.02 (0.76, 1.35)	0.98 (0.68, 1.41)	1.44 (0.79, 2.64)	1.02 (0.77, 1.36)
**PREGNANCY HISTORY**						
Parity
Nulliparous	Reference
One child	1.08 (0.79, 1.47)	1.02 (0.62, 1.70)	1.10 (0.86, 1.40)	1.01 (0.73, 1.38)	1.01 (0.60, 1.71)	1.03 (0.80, 1.33)
Two children	0.88 (0.59, 1.31)	0.77 (0.39, 1.52)	0.95 (0.70, 1.29)	0.82 (0.54, 1.24)	0.67 (0.32, 1.39)	0.87 (0.63, 1.21)
Three or more children	0.78 (0.49, 1.25)	0.95 (0.47, 1.94)	0.90 (0.63, 1.27)	0.70 (0.43, 1.16)	0.84 (0.39, 1.80)	0.82 (0.56, 1.19)
Prenatal care began in
First trimester	Reference
After first trimester/no care	1.01 (0.72, 1.41)	1.62 (0.98, 2.67)	1.17 (0.91, 1.50)	1.17 (0.83, 1.65)	1.71 (1.02, 2.87)	1.30 (1.00, 1.68)
**PATERNAL CHARACTERISTICS**						
Paternal age at child birth
<20	0.63 (0.26, 1.55)	1.49 (0.52, 4.24)	0.79 (0.42, 1.50)	0.67 (0.27, 1.67)	1.45 (0.50, 4.23)	0.82 (0.43, 1.58)
20–29	Reference
30–34	1.06 (0.75, 1.49)	1.17 (0.66, 2.06)	1.13 (0.86, 1.47)	1.08 (0.75, 1.55)	1.26 (0.69, 2.31)	1.16 (0.88, 1.54)
≥35	1.28 (0.92, 1.77)	1.31 (0.76, 2.27)	1.27 (0.98, 1.65)	1.31 (0.92, 1.87)	1.45 (0.80, 2.63)	1.32 (1.00, 1.75)
Per 1-year increase	1.02 (1.00, 1.04)	1.02 (0.99, 1.05)	1.02 (1.00, 1.03)	1.02 (1.00, 1.04)	1.02 (0.99, 1.06)	1.02 (1.00, 1.04)

*^a^Adjusted for birthyear. ^b^Adjusted for the following variables: birth year, child sex, plurality, maternal race/ethnicity, maternal age, parity, and prenatal care. Prenatal care not included in the model for child’s sex*.

## Discussion

We found a statistically non-significant association between high birth weight and RMS, even though our models suggested small (21–28%) risk increases, particularly for embryonal RMS, among children in the 4000–5250 g birth weight group. Positive associations were suggested for birth weight with all of the measures we employed and embryonal RMS. No associations were apparent for alveolar RMS and our measures of fetal size at birth. While statistically non-significant, our point estimates for embryonal RMS group are similar in direction to those reported by Ognjanovic et al. (14), the study that has some sample overlap with our study (about one-third). However, due to the smaller number of cases in our study our estimates are less precise. Apart from birth weight, we also assessed fetal growth as measured by term birth only, birth weight adjusted for gestational age, and large for gestational age to examine whether an increased fetal growth is more important than the absolute birthweight reached. However, we were unable to provide evidence for either measure being relevant.

The genetic aberrations observed among embryonal RMS and alveolar RMS are associated with both genetic and epigenetic changes resulting in overexpression of genes in the *IGF2* pathway (such as *IGF2* and *IGFR1*). Accelerated fetal growth, often measured as high birth weight for gestational age, can be a marker of such changes in DNA structure. However, many different factors determine birth weight including parental build, race/ethnicity, and diet. Therefore, the fetal growth measures based on birth weight and gestational age are at best imprecise markers of overgrowth disorders linked to aberrations in the *IGF2* pathway and also implicated in RMS development. Thus, it is perhaps more important to either determine a precise marker or to examine factors that may trigger molecular changes of the genes in the *IGF2* pathway leading to increased susceptibility for childhood RMS. Studies examining associations between environmental factors and epigenetic changes in *IGF2* linked to various cancers are lacking. So far, only a few studies examined associations between preconceptional as well as pre- and early post-natal factors for RMS. These studies reported positive associations for X-ray exposure during pregnancy, parental preconceptional use of marijuana and cocaine, maternal antibiotic use during pregnancy, organ meat consumption by children, childhood infections, and childhood exposure to chemicals (16, 17, 24). However, one study was very small (33 cases and 99 controls, 24), and two reports were on the same sample (322 cases and 322 controls matched on race, sex, and age) and not only measured exposures retrospectively but required mothers to recall exposures from 8 to 9 years prior to the interview (16, 17).

Our findings on male sex and per year increase in parental age at child’s birth for all RMS types are consistent with previous studies (14, 24–26). In addition, our data showed a moderate (1.7-fold) increase in risk for alveolar RMS among those with no or late prenatal care and a 1.3-fold increase in risk for all-type RMS among children with ≥35-year-old fathers at child birth, which have not been reported before. Absence of early prenatal care is often associated with mothers with lower SES status characterized by lower income, less education, being of certain racial/ethnic groups, and with unintended pregnancies (27, 28). Furthermore, a recent study of non-pregnant US women ages 15–44 reported that both non-white and lower SES women are less likely to meet the recommended daily intake of ≥400 μg folic acid (29). Consistent with these findings, we observed more mothers among the lowest census-based SES group (24%), Hispanic and other non-white mothers (25% and 19% respectively) reporting later/no prenatal care compared to the highest SES group (10%), or non-Hispanic white mothers (13%). This difference in report of later/no prenatal care across the two SES groups were higher among the cases (32 vs. 13% in lowest vs. highest SES group) compared to controls (24 vs.10% in lowest vs. highest SES group). Similarly, the differences in prenatal care across the two racial/ethnic groups were even more notable among the alveolar RMS cases (36% among Hispanic and 37% among other non-white mothers vs. 4% among white mothers, respectively) compared to controls (25 vs. 13%) and the embryonal RMS cases (21 vs. 17%). However, despite these varying distributions in timing of prenatal care by SES and race/ethnicity, we did not observe significant interactions between prenatal care and SES (*p* = 0.15) or maternal race/ethnicity (*p* = 0.15). The positive association we observed with alveolar RMS remained even in the model adjusted for both maternal race/ethnicity and SES [OR (95% CI): 1.70 (1.02, 2.83)]. Lack of early prenatal care may be associated with lack of important nutrients in the maternal diet, exposure to harmful agents, or risky lifestyle factors, all of which should be explored in future studies.

We conducted a population-based case-control study with controls selected at random from all California born children. Thus, our study is less likely to have been affected by bias in control selection. Furthermore, we were able to link our data to death records and exclude controls who died prior to the diagnosis of the frequency matched cases, reducing potential inaccuracy in estimates. RMS is a rare disease, but our data for a 20-year period made it possible not only to use a uniform case/control ascertainment and examine various risk factors for RMS, but also to distinguish the two most common histological subtypes. Embryonal and alveolar RMS have distinct genetic characterizations, age- and sex-specific distributions, and different prognoses (1, 2]. Thus, it is likely that different factors play an etiologic role for these subtypes, which was supported by the differences we observed across the two subtypes for risk factors of interest such as fetal growth measures, prenatal care, and maternal race/ethnicity.

Our study was limited by multiple comparisons and potential measurement errors associated with birth certificate data. While the use of California birth certificates for all risk factors allowed us to avoid recall bias and provided fairly complete data, we were limited in terms of the types of data available to assess accelerated fetal growth. We used large/small for gestational age, term birth weight, and birth weight adjusted for gestational age as measures of accelerated fetal growth whereas fundal height or repeated ultrasound may be more accurate measures, which were not available to us. Furthermore, data quality may not be equal for all variables of interest. For example, slightly more birth records missed or had implausible values for gestational age data (4%) compared to most other variables including birth weight, which may introduce selection bias if the missing data are non-random. Higher proportions of missing or inaccurate gestational age data on birth records have been reported for women of lower SES status, receiving later prenatal care, and non-white mothers (30, 31). In our data, we found this to be true of missing data for census-based SES and maternal race/ethnicity, but not for prenatal care, and there was no difference in missing data by case status. Studies on reliability and validity of birth certificate data have indicated high reliability and validity for information on demographics, birth weight, delivery method, and source of prenatal care payment and low to moderate for gestational age and prenatal care data (32–34). The gestational age information we used was estimated based on date of last menstrual period. This method is also subject to inaccuracies for mothers who have an irregular menstrual cycle, bleeding in early pregnancy, or preconceptional amenorrhea (35, 36), and may introduce additional errors in fetal growth measurements.

In summary, our study provides some additional evidence in support of a weak association between accelerated fetal growth and childhood RMS. Furthermore, the estimated effect size reflects those from earlier reports for the embryonal subtype. However, sample size limitations preclude us from drawing conclusions except that the effect seems to be relatively weak. Given the sample size for the embryonal subtype and controls in this study, the smallest effect size the study can detect at 80% power is an OR of 1.40. Our findings on starting time of prenatal care and parental age at childbirth further support the notion that the early fetal/embryonic environment may be important for developing RMS in early childhood. However, further study with better measures of prenatal care is needed to explore and confirm this association. Given that the majority of RMS cases are sporadic in nature, environmental factors may play an important role in the genomic and epigenetic alterations causing these tumors. For example, diet and supplement intake during pregnancy may cause molecular alterations observed in RMS cases. Future studies should incorporate additional, accurate measures of early life and pregnancy exposures associated with birth weight or have more accurate fetal growth measure such as fundal height and additional predictors of birth weight (e.g., parental height and weight, pregnancy weight gain, diet/supplements) to improve our understanding of RMS etiology.

## Authors Contribution

Anshu Shrestha designed the study, analyzed and interpreted the data, and drafted the manuscript. Beate Ritz, Julia E Heck, and Michelle Wilhelm designed the study, provided critical input on each step of the study, guided analysis and interpretation of the data, and revised the manuscript. Simona Ognjanovic provided critical input in analysis of the data and revised the manuscript. Christina A. Lombardi contributed to data analysis and revised the manuscript.

## Conflict of Interest Statement

The authors declare that the research was conducted in the absence of any commercial or financial relationships that could be construed as a potential conflict of interest.
